# Fertility preservation by ex vivo oocyte retrieval in a 17-year-old with bilateral borderline ovarian tumors: a case report

**DOI:** 10.5935/1518-0557.20260011

**Published:** 2026

**Authors:** Rodrigo Guimarães Furtado, Francisco Furtado, Reitan Ribeiro, Ivana Rippel Hauer, Luiza Sonaglio, Fabricio Furtado, Gustavo Wandresen, Renato Nisihara

**Affiliations:** 1 Fertway Reprodução Humana - Curitiba, PR, Brazil

**Keywords:** fertility preservation, ex vivo oocyte retrieval, ovarian tumor, adolescent, cryopreservation

## Abstract

We report a case of fertility preservation through ex vivo oocyte aspiration in an adolescent who underwent bilateral oophorectomy due to asynchronous borderline ovarian tumors. A 17-year-old nulligravid patient was initially diagnosed with a left-sided serous borderline ovarian tumor and treated with left salpingo-oophorectomy. One year later, a lesion was identified in the contralateral ovary, with surgical indication for salpingo-oophorectomy. Ovarian stimulation was performed using recombinant FSH and hMG, with final oocyte maturation triggered by a GnRH agonist. Oocyte retrieval was conducted ex vivo, immediately after surgical removal of the ovary. A total of 13 oocytes were obtained, 10 of which were in metaphase II and successfully cryopreserved. This case highlights the feasibility and efficacy of ex vivo oocyte retrieval as a fertility preservation strategy in oncology patients requiring urgent bilateral oophorectomy.

## INTRODUCTION

Borderline ovarian tumors (BOTs) represent a distinct subset of epithelial ovarian tumors, classified between benign and malignant lesions, characterized by low malignant potential. Early clinical diagnosis is often challenging due to typically subtle initial symptoms (Chen *et al*., 2024). BOTs account for approximately 10% to 20% of epithelial ovarian tumors, making them one of the most common gynecological neoplasms. Although generally slow-growing, these tumors have the capacity to spread to other parts of the body and, over time, may progress to invasive cancer. Six histological subtypes of BOTs are recognized-serous, mucinous, seromucinous, endometrioid, clear cell, and Brenner tumors-with serous and mucinous types being the most prevalent (Zilliox *et al*., 2021). This neoplasm frequently affects young patients and is bilateral in approximately 10% of cases, which poses a significant challenge for fertility preservation (Chen *et al*., 2024).

Ex vivo oocyte retrieval is an innovative technique that, to date, has been reported only six times in the literature and has shown promise in oncologic contexts requiring oophorectomy. The procedure involves follicular aspiration directly from the freshly resected ovary following controlled ovarian stimulation. This approach enables the retrieval of viable gametes, particularly in situations where transvaginal access is contraindicated due to the risk of tumor cell dissemination. Although still rarely performed, the technique has shown encouraging results in selected cases, especially among young patients with preserved reproductive potential. Its application may broaden future motherhood possibilities for women with gynecologic cancers (Bocca *et al*., 2011; de Carvalho *et al*., 2024; Fatemi *et al*., 2011; Higuchi *et al*., 2024).

## CASE DESCRIPTION

A 17-year-old female patient, non-smoker, nulligravid, with regular menstrual cycles and no comorbidities, was referred by the oncology team at the Erasto Gaertner Hospital-a regional cancer treatment center located in Curitiba, Brazil-for fertility preservation counseling at our clinic.

Menarche occurred at age 11, with 28-day cycles, 7 days of menstrual flow, and mild dysmenorrhea. The patient had no history of chemotherapy, radiotherapy, hormonal treatments, or genetic disorders. Family history included a maternal grandmother with breast cancer and a father and uncle with skin cancer. Her anti-Müllerian hormone (AMH) level was 0.86 ng/mL, and her blood type was O Rh-negative.

At age 16, she was diagnosed with a left-sided serous borderline ovarian tumor and underwent left salpingo-oophorectomy, right ovarioplasty, omentectomy, and peritoneal biopsies via laparoscopy on August 16, 2023. Histopathological analysis confirmed serous borderline ovarian tumors in both ovaries. The right ovary contained a 3.5 × 3.3 cm tumor with an intact capsule and no surface involvement. The left ovary harbored a 9.0 × 7.0 cm multilocular tumor with internal papillary projections, also with an intact capsule and no surface or adjacent structure involvement. Biopsies from the omentum, paracolic gutters, pouch of Douglas, diaphragms, left fallopian tube, and other sampled structures showed no evidence of neoplasia or tumor dissemination. These findings were consistent with disease confined to the ovaries, without stromal invasion or metastatic spread in the evaluated samples.

At that time, the decision was made to perform cystectomy of the right ovary, preserving the ipsilateral ovarian tissue, as there was no evidence of surface involvement. The patient was subsequently monitored as an outpatient, with routine follow-up visits and imaging every 90 days.

In May 2024, a follow-up pelvic MRI identified two cysts (2.8 cm and 1.2 cm) in the right ovary, with an overall ovarian volume of 25cm^3^. The uterus measured 99 cm^3^ in volume. Given the suspicion of a new contralateral borderline ovarian tumor, the oncology team recommended right salpingo-oophorectomy (with uterine preservation). Due to the patient’s young age, high likelihood of cure, and future desire for pregnancy, she was referred again for fertility preservation assessment.

Following multidisciplinary evaluation, a controlled ovarian stimulation followed by ex vivo oocyte retrieval during the scheduled oophorectomy was proposed. Transvaginal ultrasound-guided follicular aspiration was contraindicated due to the risk of peritoneal tumor dissemination from potential rupture of neoplastic cysts during intracavitary puncture.

Ovarian stimulation started on July 17, 2024, 2 days after the patient’s period. The patient received follitropin delta (Rekovelle^®^) at 10 µg/day combined with menotropin (Menopur^®^) at 150 IU/day for seven days. From stimulation day 8 onward, the Menopur dose was increased to 300 IU/day, while the Rekovelle dose remained unchanged until day 12. Dydrogesterone (Duphaston^®^) at 20 mg/day orally was initiated on the first day of stimulation for LH surge suppression, following the Progestin-Primed Ovarian Stimulation (PPOS) protocol. On stimulation day 11, eight follicles measuring between 20 and 23 mm were observed, and final oocyte maturation was triggered on day 12 with triptorelin 0.3 mg (Gonapeptyl^®^).

Due to the presence of an imperforate hymen, transvaginal ultrasound monitoring was not feasible. Follicular assessment was therefore performed transabdominally, which may account for the discrepancy between the number of follicles visualized and the number of oocytes retrieved, given the lower resolution of this approach. Oocyte retrieval was performed ex vivo, concomitant with the scheduled bilateral oophorectomy, 36 hours after the trigger.

The combination of follitropin delta and hMG is supported by the multicenter MARCS study, which demonstrated a higher number of oocytes retrieved and good-quality blastocysts compared to recombinant gonadotropin alone (Bissonnette *et al*., 2021). The use of dydrogesterone for LH suppression in PPOS is backed by a randomized clinical trial by Yu *et al*. (2018), showing comparable efficacy to medroxyprogesterone acetate and favorable reproductive outcomes.

Two days later, the patient underwent right salpingo-oophorectomy via laparoscopy. The ovary was extracted through a small suprapubic Pfannenstiel incision, protected within a surgical plastic retrieval bag, with ligation of the infundibulopelvic vessels performed 30 seconds before ovary removal.

**Figure 1:**
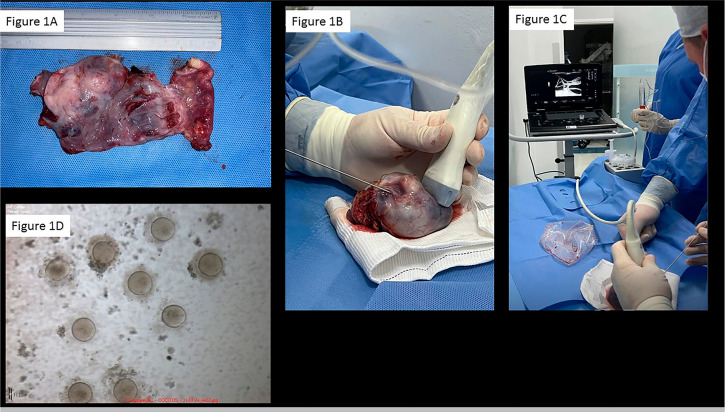
A. Right ovary after ex vivo follicular aspiration. B. Ex vivo ultrasound-guided aspiration of the right ovary. C. Ex vivo ultrasound-guided aspiration of the right ovary - follicular fluid visible on the ultrasound screen. D. Nine of the ten mature oocytes after denudation.

The surgical specimen (Figure 1A) was kept within the plastic bag immersed in warmed saline solution at 37°C. The first follicular puncture started approximately one minute after ovary removal and was performed under ultrasound guidance (Figure 1B). Aspiration was conducted without complications, including collection of the hematic fluid present inside the retrieval bag.

The follicular fluid was immediately transferred to sterile, pre-warmed microtubes containing the following solution: 90% Multipurpose Handling Medium Complete (MHM-C) with gentamicin and 10% INGÁMED Serum, a protein supplement for cell and tissue culture.

The tubes were placed in a TO 42^®^ (WTA - Watanabe Tecnologia Aplicada, São Paulo) portable oocyte transport device with active electronic temperature control (Figure 1C). Transport from the operating room to the IVF laboratory took 18 minutes, with continuous temperature maintenance at 37°C±0.5°C throughout transit, as monitored by the device.

Upon arrival at the laboratory, the material was immediately processed without incident. Post-procedure analysis confirmed the retrieval of 13 oocytes, 10 of which were at metaphase II (mature) and 3 atretic. All mature oocytes were successfully vitrified and properly stored.

## DISCUSSION

This case report describes an innovative fertility preservation strategy using ex vivo oocyte retrieval after controlled ovarian stimulation in a 17-year-old undergoing bilateral oophorectomy for borderline ovarian tumors. Multidisciplinary planning, precise execution, and a temperature-controlled transport protocol demonstrated the feasibility and safety of the technique, even with a slightly reduced ovarian reserve. The retrieval of 10 mature oocytes highlights its clinical relevance in urgent oncologic settings.

As illustrated in this case, women diagnosed with cancer during their reproductive years require comprehensive fertility care, given the gonadotoxic potential of oncologic treatments (Massarotti *et al*., 2021). This case reinforces the critical importance of fertility preservation counseling, offering women undergoing cancer treatment the possibility of future childbearing. Effective communication and collaboration between oncology and reproductive medicine services are essential to facilitate timely patient referral (Barioni & Gozzo, 2024).

Direct aspiration of the ovary immediately after surgical resection, although infrequent, represents a valuable alternative in specific clinical scenarios-such as when transvaginal access is contraindicated or, as in this report, in the presence of an increased risk of tumor cell spillage from follicular fluid during transvaginal ultrasound-guided oocyte retrieval (Bocca *et al*., 2011). The use of ex vivo oocyte retrieval can serve as a fertility preservation strategy for patients requiring bilateral oophorectomy due to asynchronous borderline ovarian tumors (Fatemi *et al*., 2011).

To date, seven cases have been reported worldwide, with details summarized in the Table 1. In the Brazilian experience, only one previously reported case of ex vivo ovarian oocyte retrieval was found (de Carvalho *et al*., 2021). The first successful pregnancy and live birth following this technique was reported in Brazil in 2024, after the retrieval of 16 mature oocytes and 3 in vitro matured oocytes from a 28-year-old patient with bilateral BOT (de Carvalho *et al*., 2024). In the case presented involves the youngest patient ever described in the literature to undergo this procedure.

**Table 1:** Summary of published cases of ex vivo oocyte retrieval following ovarian stimulation in patients with ovarian tumors.

Reference	Age	Marital Status / Parity / Clinical History	COS Protocol	Type of Surgery	Pathology	Ultrasound-Guided Retrieval	Oocytes Retrieved	Oocytes Matured In Vitro
Fatemi *et al*. (2011)	27	Not reported; nulligravid; previous laparoscopic left salpingo-oophorectomy; recurrent serous papillary adenocarcinoma	rFSH 200 IU/day; ganirelix 0.25mg/day from day 6; trigger with urinary hCG 10,000IU	Laparotomy	Recurrent serous papillary adenocarcinoma	No	13	0
Bocca *et al*. (2011)	25	Single; nulligravid; previous laparoscopic salpingo-oophorectomy; serous borderline tumor	rFSH 200 IU/day; ganirelix 0.25mg/day from day 7 to 10; trigger with rhCG 250µg on day 10	Laparoscopy (~34-35h after trigger)	Serous borderline tumor	No	14	0
Pereira *et al*. (2017)	37	Single; nulligravid; AFC ~14	rFSH 300IU/day + hphMG 150IU/day + letrozole; then rFSH 150IU/day; ganirelix 0.25 mg/day; trigger with rhCG 250 µg on day 12	Laparotomy (~34h after trigger)	Not reported	No	7	0
de la Blanca *et al*. (2018)	31	Single; nulligravid; previous left salpingo-oophorectomy; mature teratoma; AMH 1.1 ng/mL	Corifollitropin alfa 150 µg; rFSH 200IU/day; ganirelix 0.25 mg/day; trigger with rhCG 250 µg on day 10	Laparoscopy (~35 hafter trigger)	Struma ovarii	Yes	5	0
Rezacova *et al*. (2021)	34	Single; nulligravid; no family history of cancer; micropapillary serous BOT; AMH 3.91 ng/mL	recFSH (Puregon 175IU/day); Cetrotide from day 6; Ovitrelle on day 11	Laparotomy (~35h after trigger)	Micropapillary serous borderline tumor	No	10	9
de Carvalho *et al*. (2021)	28	Married; nulligravid; bilateral serous BOT; elevated CA-125; risk of dissemination	Corifollitropin alfa 150 µg + rFSH 250IU/day + ganirelix; trigger with triptorelin	Laparoscopy (~37h after trigger)	Bilateral serous borderline tumor with microinvasion	Yes	16	3

In other studies, oocyte retrieval and live birth outcomes from surgically excised ovarian tissue followed by in vitro maturation (IVM) have been described without prior ovarian stimulation (Higuchi *et al*., 2024; Segers *et al*., 2015). Due to the technical complexity of IVM, our team opted against ex vivo oocyte retrieval without prior stimulation and IVM.

This case also highlights the critical importance of strict standardization of the oocyte transport protocol. The use of a portable device with active electronic temperature control, maintained at 37±0.5°C, was essential to ensure gamete integrity during transport from the operating room to the IVF laboratory.

Every step of the process must be meticulously monitored. In this case, the chosen ovarian stimulation protocol was successfully implemented, and despite a slightly reduced ovarian reserve, the patient exhibited a satisfactory response to stimulation. Given the high risk of infertility resulting from oncologic treatments, the integration of reproductive medicine into the oncologic care of young patients has gained increasing relevance. Fertility preservation has become an essential component of cancer care, requiring a multidisciplinary approach and active engagement in educating both patients and physicians about the available options (Barioni & Gozzo, 2024).

The patient, aged 17 years, yielded 10 mature (MII) oocytes following stimulation. Although current literature lacks robust data on euploidy rates in adolescents, younger patients are known to have significantly lower aneuploidy rates. According to Namath *et al*. (2025), women under 35 years require, on average, 15 mature oocytes to obtain three euploid blastocysts with a 70% probability. However, with 10 MII oocytes, patients in this age group still have over a 75% chance of achieving at least one live birth. Given the patient’s exceptionally young age and the high expected euploidy rate, the recovery of 10 mature oocytes is likely to provide a meaningful opportunity for future biological parenthood.

## CONCLUSION

This case demonstrates that the use of ex vivo oocyte retrieval enabled fertility preservation in an adolescent patient undergoing bilateral oophorectomy for asynchronous borderline ovarian tumors is feasible and effective. In addition, even in emergent oncologic scenarios involving very young patients is safe. The recovery of ten mature oocytes, in a context of low expected aneuploidy, offers a realistic chance of future biological motherhood. This report underscores the importance of individualized fertility preservation planning and highlights the need for timely, coordinated efforts between oncology and reproductive medicine teams to expand fertility options for adolescent cancer patients.
